# Supplementary material for: COZOID: contact zone identifier for visual analysis of protein-protein interactions
Source: BMC Bioinformatics. 2018 Apr 6;19:125. doi: 10.1186/s12859-018-2113-6 (PMC5889581; doi:10.1186/s12859-018-2113-6)
Supplement: Supplementary file 2 — Software build. Executable binary file of the software tool. (ZIP 118,784 kb) [file 12859_2018_2113_MOESM2_ESM.zip › cozoid/java/docs/beansbinding-1.2.1-doc.zip!/org/jdesktop/el/impl/lang/package-tree.html]

org.jdesktop.el.impl.lang Class Hierarchy (Beans Binding)


---


|  |  |  |
| --- | --- | --- |
| **Overview** \| **Package** \| Class \| Use \| **Tree** \| **Deprecated** \| **Index** \| **Help** | |  |
| **PREV**   **NEXT** | **FRAMES**    **NO FRAMES**     **All Classes** |  |


---


## Hierarchy For Package org.jdesktop.el.impl.lang

**Package Hierarchies:**: All Packages

---

## Class Hierarchy

- java.lang.Object
  - org.jdesktop.el.impl.lang.**ELArithmetic**
    - org.jdesktop.el.impl.lang.**ELArithmetic.BigDecimalDelegate**- org.jdesktop.el.impl.lang.**ELArithmetic.BigIntegerDelegate**- org.jdesktop.el.impl.lang.**ELArithmetic.DoubleDelegate**- org.jdesktop.el.impl.lang.**ELArithmetic.LongDelegate**
    - org.jdesktop.el.**ELContext**
      - org.jdesktop.el.impl.lang.**EvaluationContext**
      - org.jdesktop.el.impl.lang.**ELSupport**- org.jdesktop.el.impl.lang.**ExpressionBuilder** (implements org.jdesktop.el.impl.parser.NodeVisitor)- org.jdesktop.el.**FunctionMapper**
            - org.jdesktop.el.impl.lang.**FunctionMapperFactory**- org.jdesktop.el.impl.lang.**FunctionMapperImpl** (implements java.io.Externalizable)
            - org.jdesktop.el.impl.lang.**FunctionMapperImpl.Function** (implements java.io.Externalizable)- org.jdesktop.el.**VariableMapper**
                - org.jdesktop.el.impl.lang.**VariableMapperFactory**- org.jdesktop.el.impl.lang.**VariableMapperImpl** (implements java.io.Externalizable)

---


|  |  |  |
| --- | --- | --- |
| **Overview** \| **Package** \| Class \| Use \| **Tree** \| **Deprecated** \| **Index** \| **Help** | |  |
| **PREV**   **NEXT** | **FRAMES**    **NO FRAMES**     **All Classes** |  |


---
